# Diagnostic Procedures for Inflammatory Bowel Disease: Laboratory, Endoscopy, Pathology, Imaging, and Beyond

**DOI:** 10.3390/diagnostics14131384

**Published:** 2024-06-28

**Authors:** Seung Min Hong, Dong Hoon Baek

**Affiliations:** Department of Internal Medicine, Pusan National University School of Medicine and Biomedical Research Institute, Pusan National University Hospital, Busan 49241, Republic of Korea; lucky77i@naver.com

**Keywords:** inflammatory bowel disease, diagnostic procedures, review

## Abstract

Diagnosing inflammatory bowel disease (IBD) can often be challenging, and differentiating between Crohn’s disease and ulcerative colitis can be particularly difficult. Diagnostic procedures for IBD include laboratory tests, endoscopy, pathological tests, and imaging tests. Serological and stool tests can be easily performed in an outpatient setting and provide critical diagnostic clues. Although endoscopy is an invasive procedure, it offers essential diagnostic information and allows for tissue biopsy and therapeutic procedures. Video capsule endoscopy and device-assisted enteroscopy are endoscopic procedures used to evaluate the small bowel. In addition to endoscopy, magnetic resonance imaging, computed tomography, and ultrasound (US) are valuable tools for small bowel assessment. Among these, US is noninvasive and easily utilized, making its use highly practical in daily clinical practice. Endoscopic biopsy aids in the diagnosis of IBD and is crucial for assessing the histological activity of the disease, facilitating a thorough evaluation of disease remission, and aiding in the development of treatment strategies. Recent advances in artificial intelligence hold promise for enhancing various aspects of IBD management, including diagnosis, monitoring, and precision medicine. This review compiles current procedures and promising future tools for the diagnosis of IBD, providing comprehensive insights.

## 1. Introduction

Inflammatory bowel disease (IBD) primarily includes ulcerative colitis (UC) and Crohn’s disease (CD). The global number of IBD patients increased sharply from 3.3 million in 1990 to 4.9 million in 2019, leading to an increased disease burden [1]. Additionally, according to the 2020 IBD fact sheet in Korea by the Korean Association for the Study of Intestinal Diseases (KASID), the prevalence of CD in Korea was 36.9 per 100,000 population in 2019, and the prevalence of UC was 65.7 per 100,000 population in the same year, showing a significant increase compared with the past [2]. The exact etiology of IBD remains unclear, but it is thought to result from a complex interaction between genetic factors, environmental triggers, disease cofactors, intestinal microflora, and mucosal immunity in patients [3].

IBD is characterized by chronic inflammation of the gastrointestinal (GI) tract, with CD potentially affecting the entire digestive tract from the mouth to the anus, whereas UC is confined to the colorectum. UC generally causes inflammation primarily in the mucosal layer, whereas CD causes transmural inflammation that affects the entire bowel wall [4]. If the disease activity of IBD is inadequately controlled, it can cause abdominal pain, diarrhea, GI bleeding, fatigue, and underweight. Severe cases can lead to complications such as strictures, obstructions, perforations, fistulas, abscesses, and an increased risk of cancer [4,5]. Patients with IBD are at a higher risk of cancer, which can be attributed not only to chronic inflammation but also to treatments for IBD [6]. Patients with IBD have an increased risk of colorectal cancer, small bowel cancer, intestinal lymphoma, and cholangiocarcinoma. The use of thiopurine and anti-tumor necrosis factor-α (anti-TNF-α) drugs further increases the risk of non-melanoma skin cancer (NMSC) and lymphoma [6]. Furthermore, IBD can be accompanied by extra-intestinal manifestations in the musculoskeletal system, skin, and eyes [7]. Currently, there is no complete cure for IBD, which significantly affects patients’ quality of life, mental health, work productivity, and healthcare resources, making it a crucial disease to address [8,9].

IBD is suspected when symptoms and typical endoscopic or radiological findings are observed. However, the diagnosis of IBD is challenging because of the lack of standardized diagnostic tools. Additionally, symptoms may not always be typical and may be similar to other conditions, such as irritable bowel syndrome (IBS), leading to diagnostic delays [10]. Literature reports that the diagnostic delay for IBD ranges from 2 months to 8 years. UC tends to have a shorter diagnostic delay than CD, as UC is confined to the colorectum and patients are more likely to present with rectal bleeding, whereas CD symptoms can be vague, leading to a longer diagnostic delay [10,11,12]. Additionally, approximately 5–15% of patients with IBD are classified as having indeterminate colitis, where it is not possible to determine whether it is CD or UC [13]. A delayed diagnosis is associated with an increased need for surgery and a poor prognosis due to a poor response to drug therapy [14,15]. Therefore, the correct diagnosis of IBD is as crucial as its treatment.

Diagnostic approaches for IBD have gradually changed over the past few decades, evolving into comprehensive methods that include clinical symptoms, laboratory tests, endoscopy, imaging, histological examinations, and artificial intelligence (AI). This re-view covers various diagnostic procedures for IBD.

## 2. Diagnostic Procedures for Inflammatory Bowel Disease

### 2.1. Laboratory Tests

#### 2.1.1. Serologic Tests

##### C-Reactive Protein

Laboratory tests are the least invasive methods for diagnosing IBD, and in clinical practice, serological tests and stool tests are mainly used. Since there is no single diagnostic tool for IBD, clinical physicians perform various tests and combine the results with clinical findings to diagnose IBD [16]. Serological tests and stool tests are useful for diagnosing IBD and monitoring its course. Blood sampling is invasive because it involves inserting a needle into the patient’s vein; however, it is sufficiently acceptable as it is less invasive compared to endoscopy. Stool tests are completely non-invasive, and if a patient can tolerate the inconvenience of collecting a stool sample, they can be very useful in clinical practice.

Clinicians routinely perform blood tests on patients presenting with suspected IBD symptoms. Among the routine blood test items, those helpful for differentiating IBD include the complete blood count, C-reactive protein (CRP) level, and erythrocyte sedimentation rate (ESR), all of which are associated with inflammation. However, these markers can also be elevated due to inflammation from causes other than IBD, which limits their utility as diagnostic markers for IBD [17]. Rather, due to their ability to reflect the level of inflammation, they are useful for monitoring patients with IBD. CRP, with a half-life of approximately 19 h, reflects short-term inflammation levels better than ESR, and a CRP level of <10 mg/L indicates remission of IBD [18,19]. Clinicians aim to reduce levels of inflammatory markers, and in the ‘Treat-to-Target’ strategy for IBD treatment, CRP normalization is set as a therapeutic goal [20]. Elevated CRP levels correlate with clinical severity in CD; however, this correlation is weaker in UC, with approximately 50% of UC patients having normal CRP levels [16,21]. CRP levels do not manifest uniformly in all patients. Suk et al. reported that CRP elevation could vary among individuals due to genetic differences [22].

Although CRP has low utility as a diagnostic marker, there have been studies exploring its use for diagnosis. Poullis et al. reported that, with a cut-off value of 2.3 mg/L, CRP had a sensitivity of 100% and a specificity of 67% for differentiating IBD from functional bowel disorders [23]. Additionally, a study by Cabrera-Abreu et al. on 153 pediatric patients with suspected IBD used a screening strategy combining hemoglobin and platelet counts. This screening strategy considered a result positive if one or both criteria were abnormal, demonstrating a sensitivity of 90.8% (95% Confidence Interval [CI], 83.3–95.7%) and a specificity of 80.0% (95% CI, 65.7–89.8%). However, the authors noted that these tests are not completely reliable, and further testing was necessary if clinically suspected [24].

##### Antineutrophil Cytoplasm Antibody and Anti-*Saccharomyces cerevisiae* Antibody

Antineutrophil cytoplasmic antibody (ANCA) is an autoantibody capable of releasing lysosomes to damage blood vessels and intestinal tissues, and it can also cause tissue damage through T cell immunity [25]. ANCA is associated with autoimmune diseases such as granulomatous polyangiitis (GPA) or microscopic polyangiitis (MPA). Perinuclear antineutrophil cytoplasmic anti-body (pANCA), a specific antibody for UC, is particularly useful in distinguishing UC from CD [26]. However, not all UC patients test positive for pANCA. Reports indicate that 55% of UC patients test positive for pANCA [27]. In a meta-analysis of 60 studies, Reese et al. reported that pANCA had a sensitivity of 55.3% and a specificity of 88.5% in UC. When combined with anti-Saccharomyces cerevisiae anti-body (ASCA) negativity, the sensitivity and specificity increased to 70.3% and 93.4%, respectively, in a pediatric subgroup [28]. Wang et al. reported that the specificity of pANCA+/ASCA- for differentiating UC from CD was 94.4%, and the prevalence of pANCA was higher in moderate-to-severe UC than in mild UC [29]. Additionally, Yorulmaz et al. reported that among patients with negative pANCA, 63% had mild disease, 33% had moderate disease, and 4% had severe disease, whereas among patients with positive pANCA, 46% had mild disease, 35% had moderate disease, and 19% had severe disease (*p* = 0.027) [30]. Studies have also investigated predicting treatment response using the pANCA/ASCA combination. Infliximab is a treatment option for IBD, but it is not effective in all patients. Esters et al. analyzed serum samples from 279 CD patients before administering 5 mg/kg infliximab and found that the combination of pANCA+/ASCA- in CD patients showed a lower response rate to infliximab, although the difference was not statistically significant (*p* = 0.067) [31]. Yoshida et al. reported in a single-center retrospective study including 50 UC patients that Proteinase 3 Antineutrophil cytoplasmic antibody (PR3-ANCA) positivity was significantly associated with primary nonresponse to anti-TNF-α agents (odds ratio [OR], 19.29; 95% CI, 3.30–172.67, *p* = 0.002) [32]. Meanwhile, a study reported that ANCA status is not always constant and can change depending on the course of the disease or treatment [33].

ASCA is a direct antibody against the mannan protein of Saccharomyces cerevisiae and is known to be more specific for CD than for UC. A meta-analysis reported that the diagnostic odds ratio of ASCA for differentiating IBD patients from healthy individuals was 21.1 (95% CI, 1.8–247.3), and for differentiating CD from UC, it was 10.2 (95% CI, 7.7–13.7) [34]. Another meta-analysis reported that ASCA positivity, when combined with pANCA negativity, showed a sensitivity of 54.6% and a specificity of 92.8% for CD [28]. ASCA can be associated not only with the diagnosis but also with the clinical characteristics of the disease. In a study of 156 CD patients, ASCA and ANCA were associated with the age of disease onset. Additionally, the ASCA level in CD was associated with disease behaviors such as fibrostenosis and internal penetration [35]. Hisabe et al. showed that Japanese patients with CD had lower positivity and titers of ASCA than Western patients with CD, indicating ethnic differences. In their study, ASCA positivity was correlated with CD duration [36].

ANCA and ASCA have also been used in pediatric patients with IBD. Kim et al. evaluated the diagnostic utility of ASCA and ANCA in a study involving 229 pediatric patients with IBD. The cut-off values for ASCA IgG and IgA for differentiating CD were 32.7 U/mL and 11.9 U/mL, respectively, with a specificity of 80.0%. The positivity rates for ASCA IgG were 75.4%, 17.5%, and 60.0% in the CD, UC, and indeterminate IBD (IBD-U) groups, respectively (*p* < 0.001). PR3-ANCA positivity rates were 24.0%, 17.6%, and 0% in the UC, IBD-U, and CD groups, respectively (*p* = 0.002). The positivity rates for pANCA were 33.6%, 28.0%, and 1.4% in the IBD-U, UC, and CD groups, respectively (*p* < 0.001) [37]. In another study involving 122 pediatric patients with IBD, the pANCA positivity rate was significantly higher in the UC group (69.9%) than in the CD group (30.4%), and the positivity rates of ASCA IgA (76.2%) and ASCA IgG (94.4%) were significantly higher in the CD group than in the control group. Additionally, in the CD group, the rate of ASCA IgA positivity was higher in the group that underwent surgery than in the group that did not [38].

##### IgG Anti-Laminaribioside Carbohydrate Antibody, IgA Anti-Chitobioside Carbohydrate Antibody, and IgG Anti-Mannobioside Carbohydrate Antibody

The IgG anti-laminaribioside carbohydrate antibody (ALCA), IgA anti-chitobioside carbohydrate antibody (ACCA), and IgG anti-mannobioside carbohydrate antibody (AMCA) target components of bacterial cell walls. These antibodies are known to be associated with CD rather than UC, with ALCA and ACCA positivity rates being approximately 20–40% in CD and around 10% in UC [39]. Sladek et al. collected blood samples from 134 pediatric patients with IBD (109 CD patients, 25 UC patients), and 67 controls. The sensitivity and specificity for differentiating CD from UC were 59% and 92% for ASCA, 33% and 80% for ACCA, 22% and 100% for ALCA, and 10% and 92% for AMCA, respectively. Additionally, 75.2% of CD patients tested positive for at least one of ASCA, ALCA, ACCA, or AMCA, and 49% of ASCA-negative patients were positive for one of ALCA, ACCA, or AMCA. Furthermore, in CD patients, cumulative quartile sum analysis of ACCA, ALCA, and AMCA revealed that a higher cumulative response to glycans was associated with a higher likelihood of CD localizing to the colon [40]. In a study involving 259 adult patients with IBD (137 CD patients, 122 UC patients) and 90 controls, the sensitivity for differentiating CD from UC was 60% for ALCA, 31% for ACCA, and 50% for AMCA, with specificities of 98%, 99%, and 99%, respectively. Moreover, 89% of ASCA-positive patients were also positive for at least one of ALCA, ACCA, or AMCA, and 77% of ASCA-negative patients were positive for at least one of the three markers. ALCA and ACCA positivity were associated with significantly higher ratios of complicated disease course and Crohn’s Disease Activity Index (CDAI) > 150 compared to ALCA and ACCA negativity. Additionally, the frequency of colon involvement was significantly higher in ACCA-positive patients than in ACCA-negative patients, and AMCA-positive patients were significantly younger than AMCA-negative patients [30]. Recently, a retrospective study evaluated the diagnostic value of ASCA, ACCA, Anti-I2, and AMCA in CD, CD with intestinal tuberculosis (CD-ITB), and intestinal tuberculosis (ITB) patients. The study found that ASCA IgG levels were significantly higher in CD patients compared to those with CD-ITB (*p* = 0.0003), and AMCA levels were significantly increased in moderate to severe disease compared to inactive disease. Moreover, elevated ASCA IgG and AMCA showed a higher sensitivity for differentiating CD from controls [41].

##### Anti-Outer-Membrane Porin C Antibody

The anti-outer-membrane porin C (OmpC) antibody targets the outer membrane protein of *Escherichia coli* and is recognized for its higher specificity in CD compared to UC. The positivity rate of anti-OmpC is 55% in CD and 5–10% in UC [39]. According to Wang et al., its sensitivity, specificity, positive predictive value (PPV), and negative predictive value (NPV) for distinguishing CD from healthy controls were found to be 50.7%, 87.5%, 76.6%, and 68.8%, respectively. For differentiation between CD and tuberculosis, the sensitivity, specificity, PPV, and NPV were 50.7%, 81.0%, 90.0%, and 32.7%, respectively. When combined with pANCA+/ASCA+, anti-OmpC positivity demonstrated a sensitivity of 85.7%, specificity of 69.3%, PPV of 78.0%, and NPV of 79.2%, indicating a significant enhancement in sensitivity. Moreover, anti-OmpC positivity was notably higher in complicated CD compared to uncomplicated CD (38.5%), suggesting its potential utility in predicting disease behavior [29]. The International Pediatric Inflammatory Bowel Disease (PIBD) Ahead Program (PIBD-Ahead) systemic review and consensus statements mention that anti-OmpC positivity can predict the occurrence of structuring and penetrating complications [42].

##### Bacterial flagellin, *Pseudomonas fluorescens* Bacterial Sequence I2

Flagellin induces the production of inflammatory cytokines by interacting with B cells and Toll-like receptor 5 (TLR5) [43]. The immune response to commensal microbiota is believed to be one of the mechanisms underlying IBD development, with the immune response to CBir1 considered a contributor to complicated CD. A recent study using murine colitis models validated that the commensal CBir1 T-cell reaction can exacerbate IBD [44]. In patients with CD, serum IgG antibody responses to flagellin are elevated, and there is an increase in flagellin-specific CD4 T cells. Both B and T cell responses to multiple flagellins are associated with complicated CD [45]. The anti-flagellin antibody (anti-CBir1) is recognized to be more specific for CD than UC, with IgG anti-CBir1 positivity in 55% of CD cases and 10% of UC cases [43,46,47,48]. Furthermore, anti-CBir1 is associated with stricturing behavior, long disease duration, and postoperative recurrence in CD patients [49,50,51]. Several large prospective studies have shown that anti-CBir1 can predict stenosing or penetrating disease in pediatric CD patients [52].

*Pseudomonas fluorescens* bacterial sequence I2 (anti-I2) is a DNA sequence derived from *Pseudomonas fluorescens*, identified in 43% of CD patients. Anti-I2 is more specific for CD than UC, being identified in only 9% of UC patients [53]. According to Yao et al., anti-I2 exhibited higher sensitivity (74.6%) but lower specificity (61.8%) for CD screening compared to other markers such as ANCA, ASCA, ALCA, and AMCA. Combining anti-I2 with ASCA IgA for CD diagnosis yielded a sensitivity of 87.3% and a specificity of 54.5% with one positive marker, and a sensitivity of 54.0% and a specificity of 87.3% with two positive markers [54]. Like anti-CBir1, anti-I2 is associated with stricturing behavior, long disease duration, and postoperative recurrence in CD patients [49,50,51]. Furthermore, Jiang et al. suggested that anti-I2 could serve as a biomarker to differentiate between CD-ITB and ITB [41].

##### MicroRNA

MicroRNA (miRNA) is a group of small noncoding RNAs, approximately 20 nucleotides long, first discovered in *Caenorhabditis elegans* in 1993 [55]. These molecules target messenger RNA (mRNA) by binding to the 3’-untranslated regions, leading to mRNA degradation or inhibiting transcription [56]. miRNA plays crucial roles in various cellular processes such as cell division, apoptosis, and proliferation. It is also associated with several immune-related diseases, including IBD [57,58]. In IBD, miRNA regulates inflammation, immune responses, and microorganisms, contributing to its pathogenesis [59]. Importantly, miRNA exhibits stability in circulation, making it a suitable serologic marker. It is found not only in blood but also in urine, feces, cerebrospinal fluid, and milk [57]. miRNA holds promise for use in diagnosing and treating IBD. Sun et al. conducted a systematic review and meta-analysis on miRNA as potential biomarkers for diagnosing IBD. They reported a sensitivity of 0.80 (95% CI: 0.79–0.82), a specificity of 0.84 (95% CI: 0.82–0.86), a diagnostic odds ratio of 21.19 (95% CI: 13.90–32.31), and an area under the curve (AUC) of 0.89 for diagnosing IBD using miRNA [60]. Wu et al. were the first to conduct miRNA profiling studies on IBD. They found that in patients with active UC, miR-16, miR-21, miR-24, miR-126, miR-195, miR-23a, miR-29a, and let-7f were upregulated compared to healthy controls, while miR-192, miR-375, and miR-422b were significantly downregulated. They also demonstrated that miRNA regulates epithelial cell-derived chemokine expression in the colon [61]. Several previous studies have investigated differences in miRNA expression profiles according to IBD subtypes. Wu et al. found that five miRNAs (miR-340*, miRplus-E1271, miR-199a-5p, miR-362-3p, and miR-532-3p) were increased, and two miRNAs (miR-plus-F1065 and miR-149*) were decreased in the blood samples of patients with active CD when compared to healthy controls. In active UC, 12 miRNAs were significantly increased, and miRNA-505 was decreased compared to healthy controls. MiRs-199a-5p, -362-3p, -340*, -532-3p, and miRplus-1271 were elevated in both UC and CD. Moreover, comparing active UC and active CD, differentially expressed miRNAs were identified [62]. Research on miRNA measured in the serum of IBD patients continues to this day. It has been reported that miRNAs can be used to assess disease activity and differentiate IBD from infectious colitis [63,64].

#### 2.1.2. Stool Tests

##### Fecal Calprotectin and Lactoferrin

Fecal calprotectin (FCP), first identified in 1980, constitutes approximately 60% of soluble proteins in neutrophil cytoplasm and is also present in epithelial cells, macrophages, and monocytes. It belongs to the S-100 protein family and is a calcium- and zinc-binding protein [65,66]. Calprotectin plays crucial roles in inflammatory responses, cell differentiation, and apoptosis. In the inflammatory response, calprotectin is released by activated immune cells in response to cellular damage, with elevated levels indicating more severe inflammation [67].

In instances of infection or inflammation in the intestinal mucosa, mucosal permeability rises, facilitating the migration of various leukocytes into the intestine via chemotaxis. Furthermore, the presence of diverse bacteria in the intestine triggers the release of calprotectin from leukocytes. Consequently, intestinal inflammation leads to elevated levels of FCP [68]. One of the reasons FCP serves as an indicator of intestinal inflammation is its stability. FCP remains stable at room temperature for up to seven days and is resistant to bacterial degradation. Moreover, storing samples at 4 °C has been shown to enhance stability [69,70]. Given that fecal samples can be collected non-invasively and directly contact the intestinal mucosa, FCP is considered superior to serum calprotectin in monitoring mucosal inflammation [71].

In healthy individuals, FCP levels typically remain below 50 mcg/g [65]. However, FCP levels are not consistent across all individuals and can vary with age. Park reported an FCP concentration of 15.88 mcg/g in healthy adults under 50 years old, contrasting with 160.3 mcg/g in adults over 70 years old, suggesting the necessity for age-based adjustments in FCP cut-off values [72]. A meta-analysis including seven studies suggested a cut-off value of 50 mcg/g for diagnosing small bowel CD, with a sensitivity of 0.89, a specificity of 0.55, and a NPV of 91.8% [73]. A meta-analysis by Jung et al., including 14 studies, proposed an optimal FCP cut-off value of 100 mcg/g for screening small bowel CD, with sensitivity and specificity both at 0.73 [74]. On the other hand, the STRIDE-II recommendations mention an FCP concentration of 150–250 mcg/g as a grey zone, indicating that clinical decisions can be challenging with such ambiguous values [20]. In a study involving 870 patients, FCP levels were measured above 50 mg/dL in 85% of colorectal cancer patients, 81% of inflammatory disease patients, and 37% of patients with normal or minor endoscopic findings. Additionally, FCP demonstrated a sensitivity and NPV of 100% for detecting organic colonic diseases [75]. Petryszyn et al. conducted a meta-analysis involving 5032 IBD patients aged 16 and older, determining the sensitivity and specificity of FCP for diagnosing IBD to be 0.882 (95% CI, 0.827–0.921) and 0.799 (95% CI, 0.693–0.875), respectively [76].

Abdominal pain and diarrhea are the main symptoms of IBD, but they are also prevalent in IBS. FCP proves valuable in distinguishing between IBS and IBD. In a systematic review with a meta-analysis including 17 studies, Dajti et al. investigated the efficacy of FCP in this regard. They reported a sensitivity of 85.8% (95% CI: 78.3–91) and a specificity of 91.7% (95% CI: 84.5–95.7) for FCP in distinguishing IBS from IBD [77]. Another meta-analysis affirmed that when CRP levels are low or FCP levels are below 40 mcg/g, the likelihood of IBD is less than 1% [78]. Consequently, FCP can effectively differentiate IBS from IBD or rule out IBD, thus reducing the necessity for additional invasive tests.

Fecal lactoferrin (FL), an approximately 80 kDa iron-binding glycoprotein present in various body fluids and a significant constituent of neutrophil granules released during apoptosis, exhibits patterns similar to FCP. FL levels rise during intestinal inflammatory responses as neutrophils migrate into the intestine, making it a useful marker of inflammation levels [79]. In a meta-analysis including 10 studies, Dai et al. found FL to possess a sensitivity of 0.81 (95% CI: 0.64–0.92) and a specificity of 0.82 (95% CI: 0.61–0.93) for assessing UC activity. For CD activity, FL displayed a sensitivity of 0.82 (95% CI: 0.73–0.88) and a specificity of 0.71 (95% CI: 0.63–0.78) [80]. Another meta-analysis on the diagnostic accuracy of FL reported sensitivities and specificities of 75% and 100% for CD, and 82% and 100% for UC, indicating moderate sensitivity but high specificity in diagnosing suspected IBD [81]. Similar to FCP, FL proves useful not only in diagnosing IBD but also in monitoring the disease. A systematic review suggested that while several fecal biomarkers are challenging to substitute for FCP in clinical practice, FL shows promise as an exception. However, the review highlighted the necessity for extensive additional research for FL to become a complete alternative to FCP [82].

##### MiRNA

miRNA is found in various body fluids, including feces and blood. In fecal samples, the expression of miR-223 and miR-1246 was elevated in active IBD, while miR-16-5p was upregulated in both UC and CD patients compared to healthy controls. Additionally, miR-21-5p showed increased expression, specifically in patients with UC [83,84]. Schönauen et al. observed higher expression levels of miR-16, miR-21, miR-155, and miR-223 in fecal samples from patients with IBD compared to controls, with overall miRNA expression being higher in patients with UC than those with CD [85].

### 2.2. Endoscopic and Pathological Tests

#### 2.2.1. Endoscopic Tests

##### Lower Endoscopy

Colonoscopy stands as one of the pivotal tools for diagnosing IBD, offering not only diagnostic capabilities but also the ability to assess disease activity, treatment effectiveness, cancer surveillance, and to obtain biopsies for pathological evaluation. Additionally, it allows for therapeutic interventions such as endoscopic resection of neoplasms and dilation of strictures [53,86,87,88,89]. Given that CD often affects the terminal ileum, it is crucial to examine the terminal ileum, during a colonoscopy [90].

UC typically begins in the rectum and progresses proximally. Endoscopic features of UC include loss of vascular markings, mucosal edema, mucosal erythema, and mucosal friability. In severe cases, diffuse ulceration and spontaneous bleeding may occur [91]. In UC, inflammation is continuous and symmetrical, with skip lesions being rare. However, not all UC cases exhibit these characteristic findings. Park et al. reported that 19.2% of 240 UC patients showed an atypical lesion distribution on initial colonoscopy, with 3.3% exhibiting rectal sparing and 15.8% having skip lesions [92]. Moreover, appendiceal orifice inflammation (AOI), characterized by inflammation around the appendiceal orifice with normal surrounding mucosa, is observed in 75% of UC patients [93]. In contrast, the CD presents with aphthous ulcers, deep and large ulcerations, longitudinal ulcers, rectal sparing, segmental involvement, perianal disease, and a cobblestone appearance [94,95]. Table 1 presents a summary of the endoscopic features of UC and CD.

When diagnosing IBD via colonoscopy, it is essential not only to differentiate between UC and CD but also to distinguish IBD from infectious diseases. Infectious colitis can mimic the endoscopic findings of UC or CD but is typically an acute condition that resolves rapidly with appropriate antibiotic treatment. If clinical symptoms improve quickly with antibiotics, infectious colitis is more likely than IBD. Additionally, infectious colitis can be diagnosed through other tests, such as cultures. Conditions such as salmonellosis, shigellosis, and cytomegalovirus (CMV) colitis can present with endoscopic findings similar to those of IBD [94]. On the other hand, differentiating ITB from CD can be challenging. ITB and CD share similar symptoms, such as abdominal pain, diarrhea, hematochezia, and weight loss. Endoscopically, ITB commonly affects the ileocecal area, causing aphthous ulcers and ileocecal deformity resembling CD. However, some endoscopic features differ between ITB and CD, aiding in differentiation. ITB is more likely to present with a patulous ileocecal valve (ICV) compared to CD, generally involves fewer than four segments, and is characterized by circumferential or transverse ulcers, unlike the longitudinal ulcers seen in CD. Furthermore, ITB often exhibits inflammation around the ulcer margins, while CD typically shows normal mucosa around the ulcers. Table 2 summarizes the differential points between ITB and CD [95,96,97,98].

While CD cannot be definitively diagnosed through biopsy alone, tissue acquisition can confirm ITB. However, the diagnostic rates for ITB using tissue acid-fast bacilli (AFB), polymerase chain reaction (PCR), and culture are 40.7%, 25.7%, and 53.4%, respectively [99]. Hence, clinicians sometimes make a presumptive diagnosis based on endoscopic and other clinical findings, initiating empirical treatment. CD is treated with corticosteroids, 5-aminosalicylic acid, and immunomodulatory drugs, while ITB requires antituberculosis medications, necessitating careful judgment.

##### Upper Endoscopy

Upper GI tract involvement is not common in UC; therefore, esophagogastroduodenoscopy (EGD) is typically reserved for cases of CD. The occurrence of upper GI tract involvement in CD is relatively low, estimated at around 0.5% to 16% [100,101,102]. Due to this lower prevalence, EGD is not routinely conducted in all CD patients. However, it becomes necessary when patients present with upper GI symptoms such as nausea, vomiting, or heartburn. Moreover, in cases where the subtype of IBD is ambiguous, performing EGD can aid in diagnosing CD by detecting any upper GI tract involvement [103]. The extent of involvement varies depending on the specific regions of the upper GI tract. For instance, Sakuraba et al. found specific CD lesions in various proportions: 6.5% in the esophagus, 47.8% in the upper-to-middle stomach, 24.6% in the lower stomach, 31.9% in the duodenal bulb, and 18.1% in the second portion of the duodenum [104]. Esophageal CD involvement may manifest with erosions or multiple ulcers arranged longitudinally. These ulcers can resemble punch-out ulcers and necessitate differentiation from ulcers caused by herpes virus or CMV [105]. In the stomach and duodenum, typical presentations include erosions, ulcers, erythema, cobblestone appearance, strictures, fistulas, nodular duodenal folds, notching, and a bamboo joint-like appearance [106]. The latter is characterized by erosive fissures crossing longitudinal folds in the gastric cardia or the lesser curvature of the gastric upper body, which can be indicative of CD [107]. Routine EGD is not warranted for asymptomatic adult patients with CD. However, upper GI CD is more prevalent in children, making EGD a recommended initial evaluation for suspected IBD in pediatric patients, regardless of symptoms [108].

##### Video Capsule Endoscopy

It is not possible to visualize the entire small intestine using EGD or colonoscopy. Since 30% of CD cases are limited to the small intestine, there is a possibility of missing the diagnosis if the entire small intestine is not observed [109]. Video Capsule Endoscopy (VCE) facilitates the comprehensive visualization of the entire small intestine, and the European Society of GI Endoscopy (ESGE) recommends VCE as an initial diagnostic tool for CD [110]. Other methods for observation of the entire small bowel include enteroscopy, magnetic resonance imaging (MRI), and computed tomography (CT). However, enteroscopy is more invasive and time-consuming compared to VCE. Additionally, early or mild small bowel CD may only manifest as superficial mucosal lesions that might not be discernible on MRI or CT scans but can be observed on VCE [111]. The European Crohn’s and Colitis Organisation (ECCO) and the European Society of GI and Abdominal Radiology (ESGAR) guidelines state that the identification of three or more small bowel ulcers via VCE, without the use of non-steroidal anti-inflammatory drugs for at least one month, strongly suggests CD [16].

Several studies have demonstrated the effectiveness of VCE in diagnosing CD. One study reported that the sensitivity and specificity of VCE for diagnosing suspected small bowel CD were 93% and 84%, respectively [112]. A meta-analysis comparing the diagnostic yield of VCE with CT enterography (CTE) for suspected CD showed that VCE was superior (68% vs. 21%, *p* < 0.00001), while no significant difference was found when comparing VCE with magnetic resonance enterography (MRE) (VCE 55% vs. MRE 45%, *p* = 0.43) [113]. Another study reported that VCE was superior to MRE in detecting superficial and proximal lesions of the small bowel [114,115,116]. Additionally, a real-world study reported that VCE for CD indications resulted in positive findings in 50% of cases [117]. A recent meta-analysis evaluated the diagnostic accuracy of Pan-enteric Capsule Endoscopy (PCE) for IBD, which can observe the entire GI tract [118]. This meta-analysis showed that VCE was superior to MRE for detecting CD with an OR of 1.25 (95% CI, 0.85–1.86%). For UC, PCE had a diagnostic sensitivity and specificity of 93.8% (95% CI, 87.6–97.0%) and 69.8% (95% CI, 38.2–89.6%), respectively [118].

When performing VCE on patients suspected of having CD, it is important to consider the presence of small bowel strictures. Liquid substances can pass through some degree of stricture, so complete obstruction may not cause symptoms until later stages. Therefore, even without symptoms of obstruction, capsule retention can occur. A meta-analysis reported that the retention rates of capsule endoscopy in the overall CD cohort and suspected CD were 3.32% (95% CI, 2.62–4.2%) and 2.35% (95% CI, 1.31–4.19%), respectively [119]. In summary, VCE is a less invasive and convenient method for observing the small bowel mucosa and is useful for diagnosing small bowel CD.

##### Device-Assisted Enteroscopy

Device-assisted enteroscopy (DAE) is a method used to directly observe the mucosa of the small bowel, complementing VCE. While DAE is more invasive compared to VCE, it offers the advantage of enabling manipulation, conducting biopsies upon lesion detection, and performing therapeutic procedures such as stent placement and balloon dilation. However, less invasive tests such as VCE, MRE, and CTE are typically prioritized during the initial diagnostic stages, which may limit the utilization of DAE [120]. Generally, DAE is reserved for cases where less invasive tests do not provide sufficient information. According to the ECCO, DAE may be considered in suspected or confirmed cases of CD when a histological diagnosis is required or when therapeutic interventions such as stricture dilation, capsule retrieval, or bleeding control are necessary [121]. However, one notable limitation of DAE is its restricted availability due to the limited number of specialized centers capable of performing it. A study conducted by Lee et al. revealed an overall diagnostic yield of 78.7% for double-balloon enteroscopy (DBE) [122].

Manes et al. reported a diagnostic yield of 59.4% for CD using DBE in a high-volume center for IBD [123]. Another study indicated a diagnostic yield of 79% for suspected CD using DBE [124]. Single-balloon enteroscopy (SBE), featuring a single balloon attached to the overtube, offers easier manipulation. Despite its lower rate of complete enteroscopy compared to DBE, SBE demonstrates comparable diagnostic yields and complication rates [125,126].

#### 2.2.2. Pathological Tests

Endoscopic biopsy plays a pivotal role in distinguishing IBD from other conditions in patients suspected of having IBD [127]. When IBD is suspected, it is advisable to obtain biopsies before initiating treatment, as treatment interventions can potentially alter the mucosal morphology [128].

In the diagnostic process of IBD, it is recommended to obtain biopsies from at least two sites out of five designated areas: the right colon, transverse colon, left colon, sigmoid colon, and rectum, during ileocolonoscopy of the terminal ileum and colorectum [16,129]. For suspected CD, an endoscopic biopsy of the upper GI tract is also suggested [129]. Biopsies should be targeted in regions exhibiting active inflammation as well as in unaffected areas of the colon, particularly the rectum, to aid in an accurate diagnosis [16]. It is crucial to place the obtained tissue in separate designated containers, clearly labeled with the biopsy site, to facilitate interpretation by the pathologist. Failure to mark biopsy sites may pose challenges in distinguishing between UC and CD involvement of the colon or between small bowel CD and UC backwash ileitis. Moreover, specifying biopsy sites aids in tracking histopathological changes in different areas, establishing a diagnosis, determining treatment strategies, and monitoring treatment effectiveness [130]. A recent meta-analysis revealed that persistent histologic activity during endoscopic remission in UC was associated with higher rates of relapse [131]. Christensen et al. conducted a study involving 101 patients with CD restricted to the terminal ileum, reporting that histologic healing was associated with a reduced risk of clinical relapse, medication escalation, and corticosteroid use [132].

For microscopic diagnosis of IBD, key evaluation criteria include mucosal architecture, cellularity of the lamina propria and submucosa, infiltration of neutrophil granulocytes, and epithelial abnormalities [133]. Table 3 summarizes the microscopic features that differentiate UC from CD.

Histologically, inflammation in UC is typically continuous without skip lesions and primarily affects the mucosa, although it can occasionally involve the superficial submucosal layer. In UC, there is an abnormally high density of neutrophils, lymphocytes, and plasma cells in the lamina propria. Non-active inflammation in UC exhibits baseline features of chronic IBD, characterized by the presence of lymphocytes and plasma cells in the lamina propria [129]. Neutrophils are indicative of disease activity in UC and are present in the lamina propria, surface epithelium, and crypt epithelium during active inflammation. Neutrophil infiltration during active inflammation can lead to cryptitis, crypt abscesses, mucosal erosion, and ulcers [134,135]. Although crypt abscesses can also occur in CD, they are more frequent in UC [127]. Basal plasmacytosis, where plasma cells are concentrated in the basal layer of the lamina propria, is a hallmark finding in UC [136,137]. One study found that eosinophilia combined with basal plasmacytosis was significantly useful in diagnosing UC, with a sensitivity of 90.4% [129,138,139]. Another study reported that basal plasmacytosis was confirmed in up to 38% of IBD cases within two weeks of symptom onset and in 89% of IBD cases within 121–300 days of symptom onset, indicating a high predictive value for basal plasmacytosis [140]. Eosinophil count can vary among patients, and some studies suggest that colonic mucosal hypereosinophilia can predict non-response to medical therapy [141,142,143]. In UC, the severity of inflammatory infiltration increases towards the rectum, particularly in the lower third, which has the lowest cell density in the lamina propria [127,144]. This inflammatory infiltration diffusely expands the lamina propria [138]. Additional histological features of UC include mucosal structural abnormalities such as crypt distortion, crypt atrophy, and crypt branching, resulting from chronic inflammation and becoming more prominent with longer disease duration. Therefore, crypt architecture may appear normal in very early UC.

The most distinguishing histological features of CD compared to UC are discontinuous chronic inflammation, focal crypt architectural distortion adjacent to normal crypts, and the presence of granulomas [128,129]. Unlike UC, where inflammation is limited to the mucosa, CD extends transmurally into the submucosa and beyond and does not exhibit basal plasmacytosis, a characteristic feature of UC. However, this transmural inflammation is typically observed in resected specimens, whereas biopsy specimens are superficial, making it challenging to observe these features [138]. Histological cellularity in UC consists of mixed neutrophils, eosinophils, lymphocytes, and plasma cells. Similar to UC, the presence of neutrophils in the lamina propria, epithelium, and crypts indicates active inflammation in CD. The presence of non-caseating granulomas favors a diagnosis of CD, although this feature can also be seen in infectious diseases [128]. Moreover, non-caseating granulomas are not always present in CD, with one study reporting their presence in 15–36% of rectal biopsies [145]. Granulomas in CD have indistinct borders, and necrosis is uncommon [138]. Irregular villous architecture in terminal ileum biopsy specimens is indicative of CD. However, if continuity with the proximal colon is observed, it may suggest backwash ileitis in UC, requiring careful interpretation [128].

**Table 3:** Microscopic findings of Crohn’s disease and ulcerative colitis.

Findings	Ulcerative Colitis	Crohn’s Disease
Distribution of inflammation [128,129]	Diffuse and continuous	Patchy and focal
Involvement depth of inflammation [138]	Often mucosa, sometimes superficial submucosa	Often transmural
Crypt architectural distortion [128,129,134,135]	marked	less marked
Crypt abscesses [128,129,134,135]	Common	Scanty
Granuloma [128,129]	Rare	Present (non-caseating)
Basal plasmacytosis [136,137]	Useful for diagnosis	Not useful for diagnosis
Mucin depletion [146]	Typical	Less typical
Neuronal hyperplasia [147]	Not typical	Typical

### 2.3. Imaging Tests

Imaging tests complement or replace endoscopies in the diagnosis and evaluation of IBD. The importance of imaging is particularly emphasized in CD, which often involves the small intestine. For the endoscopic evaluation of CD, it is necessary to perform an ileocolonoscopy after bowel preparation to observe the terminal ileum. There are cases of CD in which ileocolonoscopy cannot evaluate the involved small bowel, requiring device-assisted enteroscopy or small-bowel capsule endoscopy for endoscopic evaluation. Endoscopy can be invasive, burdensome to patients during the preparation process, and sometimes accompanied by complications, making frequent performance difficult. Additionally, endoscopy may be impossible to perform depending on the patient’s clinical condition. Currently, the imaging modalities used for patients with IBD include MRI, CT, and ultrasound (US).

MRE is recommended for diagnosing CD, monitoring disease activity, evaluating therapeutic responses, and assessing perianal disease [108,148,149,150]. A meta-analysis reported that MRE had a sensitivity and specificity of 93% for diagnosing IBD [151]. Another meta-analysis reported that MRI had a sensitivity of 76%, a specificity of 91%, and an accuracy of 86% [152]. MRI also showed similar sensitivity, specificity, and accuracy in diagnosing CD to colonoscopy [153]. Grand et al. conducted a retrospective study involving 850 patients with known or suspected CD who underwent MRE. Among them, 310 underwent colonoscopy and biopsy within 90 days, and the results were compared with those of MRE. The overall sensitivity and specificity of MRE were 85% and 80%, respectively (kappa = 0.65). The sensitivity of MRI for detecting pathologically severe diseases was 87% in the terminal ileum and 88% in the colon. This study demonstrated the usefulness of MRE in evaluating CD without invasive endoscopy or biopsy [154]. A major advantage of MRE is that patients are not exposed to ionizing radiation during testing. Considering the lifelong need to monitor patients with CD, the lack of radiation exposure is a significant advantage [155]. However, the long examination time and high cost of MRI are drawbacks, and MRE requires the ingestion of a large amount of oral contrast before the test. Characteristic MRE findings in active CD include segmental mural hyperenhancement, wall thickening (>3 mm), intramural edema, stricture with upstream dilatation, ulcerations, restricted diffusion, increased mesenteric vascularity (Comb’s sign), sacculations, and reactive lymphadenopathy [156,157]. Various activity scoring systems using MRE include MaRIA, simplified MaRIA, Clermont, London, and Extended London, with MaRIA being endoscopically validated and providing the strongest evidence for clinical use [158]. Research is currently ongoing to expand the use of MRI for CD. Positron emission tomography–magnetic resonance imaging (PET-MRI) using fluorodeoxyglucose (FDG) is not yet widely used but is considered potentially useful for evaluating various inflammatory processes [159,160].

CT has advantages, such as lower cost, shorter examination time, and higher accessibility compared to MRI, but it also has the disadvantage of exposing patients to ionizing radiation. This is a significant drawback for patients requiring repeated tests. Radiation exposure is associated with increased cancer risk and non-cancer mortality [161]. A meta-analysis found that CT is less sensitive and specific than MRI for diagnosing CD [151]. A prospective study showed that CTE had the same accuracy as MRE for assessing disease activity and bowel damage in CD but was slightly inferior in identifying ileal wall enhancement and intestinal strictures [162]. The ECCO-ESGAR guidelines state that CT and MRI currently show similar performance in diagnosing CD; however, MRI should be preferred over CT, especially in young patients, owing to the ionizing radiation exposure associated with CT [16]. Common CT findings in CD include prominent bowel wall thickening (>3 mm) and mural hyperenhancement, particularly on the mesenteric side, indicating an active disease [153,163].

Intestinal US (IUS) is used as an alternative to MRI and CT because of its simplicity, low cost, and good tolerance. In addition, the US has better accessibility, allows immediate interpretation and reporting of results during the examination, and aids in quick decision-making in clinic room settings [164]. The METRIC (MR enterography or US in CD) trial, a multicenter study involving eight hospitals in the UK, compared the diagnostic accuracy of MRE and US for CD [165]. The sensitivity for detecting small-bowel disease was 97% for MRE and 92% for the US, whereas the specificity was 96% for MRE and 84% for the US. Although MRE has higher sensitivity and specificity, US has sufficiently high accuracy and advantages, leading the ECCO-ESGAR guidelines to recommend IUS for disease evaluation and monitoring [16]. Additional technologies can be integrated with the IUS. Color Doppler can be used in combination with bowel wall thickness measurement to assess disease activity. Contrast-enhanced US (CEUS) can evaluate bowel wall perfusion in real-time and is potentially useful for distinguishing fibrosis from active inflammation and assessing CD activity [153,166]. Recent studies have suggested that transmural healing in patients with CD is associated with improved long-term outcomes [167]. The IUS can be an excellent alternative for frequent evaluations or when quick decision-making regarding treatment changes is required. However, IUS results and interpretations vary among examiners. A survey conducted among members of the British Society of Gastroenterology Inflammation Bowel Disease Group revealed that many centers did not offer US services, and clinicians were less confident in making clinical decisions with US than with MRI [168].

## 3. Potential Markers and Tools

### 3.1. Biochemical Markers

#### 3.1.1. Oncostatin M

Oncostatin M (OSM), a member of the interleukin (IL)-6 cytokine family, is a pro-inflammatory cytokine produced by various immune cells [169]. OSM is also associated with the development of arthritis, dermatitis, and cancer [170]. OSM binds to glycoprotein gp130, mediating its effects, and this complex activates the OSM receptor, initiating signal transduction [171]. OSM and its receptor are more highly expressed in the intestines of patients with IBD than in healthy controls and are associated with disease severity [172,173]. Verstockt et al. reported that serum OSM was higher in first-degree relatives of patients with IBD than in control families and that OSM was upregulated in recurrent CD after surgery. Additionally, increased OSM was predictive of primary non-response to anti-TNF and vedolizumab therapy [173]. A recent meta-analysis found significantly higher OSM levels in non-responders to treatment than responders. Moreover, OSM was significantly associated with the Simple Endoscopic Score for CD, Mayo Endoscopic Score, FCP, CRP, and platelet count [174]. Although the role of OSM in IBD has not yet been fully established, it is considered a promising potential marker for the diagnosis and prognosis of IBD.

#### 3.1.2. αvβ6 Protein

The integrin αvβ6 protein is expressed on epithelial cells and is crucial in maintaining the epithelial barrier [175,176]. Kuwada et al. reported that 92.0% of patients with UC had anti-integrin αvβ6 antibodies, with a sensitivity of 92.0% and a specificity of 94.8% for diagnosing UC. [177] Another recent study found that the UC group had higher IgG anti-αvβ6 levels than the CD and IBS groups. The sensitivity of IgG anti-αvβ6 for diagnosing UC was 76.3%, with a specificity of 79.0% (vs. CD) and 96.0% (vs. IBS). IgG anti-αvβ6 levels were also associated with UC severity [178]. Livanos et al. recently reported that anti-integrin αvβ6 autoantibodies were associated with adverse outcomes related to UC up to 10 years before diagnosis [179]. These studies suggest that anti-integrin αvβ6 antibodies may be a useful biomarker for UC.

#### 3.1.3. Glycome

Proteins undergo glycosylation through post-translational modifications, and glycans are essential in various biological processes [180]. Previous studies have suggested that abnormal protein glycosylation is associated with the onset and progression of IBD [181]. Decreased glycosylation is identified in the intestinal mucus of IBD patients, which potentially leads to increased contact between bacteria and the epithelium, thereby inducing inflammation [182]. Research is ongoing on the significance of glycomes in IBD. Clerc et al. analyzed the IgA glycosylation profile of patients with IBD and healthy controls. Their model demonstrated good performance in predicting CD versus healthy controls [183]. Shubhakar et al. reported that serum N-glycomic biomarkers were associated with the prognosis of IBD in a cohort of IBD patients [184].

#### 3.1.4. Fecal Myeloperoxidase

Myeloperoxidase (MPO) is a neutrophil enzyme crucial in killing bacteria through the production of hypochlorous acid, but it can also cause inflammatory tissue damage [185]. MPO is overexpressed in many inflammatory diseases, including IBD, and fecal MPO is a potential stool biomarker [186]. A recent prospective study found that fecal MPO was significantly correlated with endoscopic activity in both CD and UC and was significantly correlated with FCP. Fecal MPO effectively predicted moderate-to-severe IBD and a complicated IBD course [187]. Subsequent studies confirmed that elevated fecal MPO levels are associated with long-term outcomes over 24 months in patients [188]. Fecal MPO has consistently shown positive research results as an IBD biomarker and is considered promising.

#### 3.1.5. Trefoil Factors

Trefoil factors (TFFs) are peptides secreted by intestinal epithelial cells and are crucial for the formation of mucosal barrier integrity [189]. Positive studies have been reported on the use of TFFs as biomarkers for UC. Patients with active UC show higher levels of TFF-3 when compared to patients with quiescent UC, CD patients, and controls, and there is a correlation with endoscopic activity [190]. Additionally, Nakov et al. reported that a TFF-3 cut-off value of 6.74 ng/mL predicted complete mucosal healing, with a sensitivity and specificity of 0.879 and 0.869, respectively [191]. In CD patients, TFF-3 exhibited a positive correlation with the Simple Endoscopic Score in pediatric patients, but not with pediatric CDAI or serum CRP. Furthermore, serum TFF-3 demonstrated 100% sensitivity and 76.2% specificity in monitoring disease activity in pediatric CD patients [192]. However, one study measured serum TFF-3 before and after anti-TNF-α induction therapy in adult CD patients and found that changes in TFF-3 did not reflect mucosal healing. Additionally, serum TFF-3 was not associated with endoscopic activity or FCP levels in adult CD patients [193].

#### 3.1.6. Leucine-Rich Alpha-2 Glycoprotein

Leucine-rich alpha-2 glycoprotein (LRG) is an inflammatory marker obtained through the proteomic screening of serum from patients with rheumatoid arthritis [194]. LRG was significantly associated with the activities of IBD and could predict mucosal healing. It was particularly effective in distinguishing endoscopically active IBD patients from those with mucosal healing, even in patients with normal CRP levels [195]. Another study also found that LRG was significantly associated with deep remission in UC patients and with disease activity in patients with normal serum CRP levels [196]. These findings suggest that LRG has the potential to be a superior serum biomarker compared to CRP.

#### 3.1.7. Serum Amyloid A

Serum amyloid A (SAA) is an acute-phase protein synthesized in the liver in response to inflammatory stimuli and is known to be involved in the immune-mediated inflammatory response of diseases such as IBD [197]. SAA is considered a potential marker for the diagnosis, assessment of disease activity, and prognosis of IBD. It has been reported that serum SAA is significantly elevated in IBD patients [198]. In a study involving 55 CD patients, SAA levels were significantly higher in patients with active disease, and showed significant correlations with the Simple Endoscopic Score and CDAI. Additionally, SAA levels demonstrated a sensitivity of 68% and a specificity of 83% for mucosal healing [199]. Wakai et al. reported that in 108 UC patients who underwent colonoscopy and were tested for CRP and serum SAA levels, SAA was significantly more associated with mucosal inflammation than CRP in patients in clinical remission (*p* < 0.01) [200].

#### 3.1.8. Dipeptidyl Peptidase-4

The role of Dipeptidyl peptidase (DPP)-4 in IBD is not yet fully understood; however, its potential as a new biomarker is being investigated. Kim et al. reported that in a large cohort of diabetic patients, those receiving DPP-4 inhibitor combination therapy had a reduced risk of autoimmune diseases—including IBD—compared to those receiving non-DPP-4 inhibitor combination therapy [201]. Additionally, Pinto-Lopes et al. demonstrated that DPP-4 could distinguish between remission and active states of IBD and predict the response to treatment [202].

#### 3.1.9. Prostaglandin E-Major Urinary Metabolite

Prostaglandin E-major urinary metabolite (PGE-MUM) levels are elevated in UC patients and have been reported as an independent predictor of histologic remission, with a sensitivity of 0.82 and a specificity of 0.82 [203]. Sakurai et al. reported significant differences in PGE-MUM levels between UC patients in endoscopic remission and non-endoscopic remission (*p* = 0.028), histologic remission and non-histologic remission (*p* = 0.004), and complete mucosal healing and non-complete mucosal healing (*p* = 0.021). Additionally, the AUCs for endoscopic healing, histologic healing, and complete mucosal healing showed no significant differences between PGE-MUM and FCP levels in UC patients [204]. The advantage of PGE-MUM is its non-invasive nature, indicating its potential as a useful biomarker.

#### 3.1.10. Melanocortin System

The melanocortin system is derived from the protein precursor pro-opiomelanocortin, comprising α-, β-, and γ-melanocyte-stimulating hormone (MSH), adrenocorticotropic hormone (ACTH), agouti- and agouti-related proteins, and their receptors, melanocortin receptors (MCRs) [205]. The melanocortin system operates through interactions between melanocortin ligands and five types of MCRs (MC1R–MC5R), which belong to the G protein-coupled receptor family [206]. MCRs are located in various parts of the body and perform functions related to melanogenesis, vascular endothelial regulation, neuroprotection, steroidogenesis, energy homeostasis, food behavior, gland secretion, and inflammation regulation, depending on the subtype. MCRs are involved in the actions of immune cells and various cytokines and chemokines, with MC1R, MC3R, and MC5R being particularly important in inflammation regulation [207].

Several biochemical actions mediated by MCRs have been thought to play a central role in IBD [208]. Maaser et al. reported that in a study on mice with dextran sodium sulfate (DSS)-induced colitis, mice with MC1R gene mutations exhibited more weight loss and more pronounced histological changes compared to C57BL/6 wild-type mice. This study was the first to demonstrate the functional role of MC1R in bowel inflammation [209]. Subsequently, Yoon et al. conducted a study in which recombinant *Lactobacillus casei* secreting α-MSH was orally administered to mice with DSS-induced colitis, resulting in the alleviation of acute colitis-related indicators (i.e., histological activity, weight loss, and MPO activity). This suggested that α-MSH, which activates MCR, could be developed as a therapeutic agent for IBD [210]. Spana et al. reported that MC1R agonists PL-8177 and PL-8331 were effective in preventing and alleviating intestinal and ocular inflammation in preclinical disease models, similar to the previously studied effects of α-MSH [211]. A recent study demonstrated that oral administration of PL-8177 to rat models with colitis induced by DSS or 2,4-dinitrobenzenesulfonic acid significantly improved colitis-related indicators compared to no treatment, highlighting the potential of PL-8177 as a therapeutic agent for gastrointestinal inflammatory diseases [212].

MC2R is primarily known for its role in steroidogenesis; however, one study suggested a potential link to colitis. Hiramoto et al. investigated the effect of ultraviolet A (UVA) and ultraviolet B (UVB) irradiation to the eyes on DSS-induced UC in mice and found that UVB eye irradiation exacerbated DSS-induced UC, with increased expression of MC2R in the colon [213].

MC3R and MC5R are also known to be involved in the inflammatory process. One study reported that activation of MC3R in mice had a protective effect against acute and delayed myocardial reperfusion injury, which was associated with reduced inflammatory markers [214]. Additionally, MC5R expressed in Ba/F3 pro-B lymphocytes is known to stimulate Janus kinase 2 (JAK2), a major pathway related to IBD, upon binding with α-MSH [215]. In a recent study including 13 patients with UC and 13 patients with CD, Gravina et al. reported that MC3R and MC5R were associated with the severity of both diseases. In addition to standard biopsies for clinical practice, the authors collected tissue samples from inflamed and normal colon mucosa for research purposes. When evaluating the expression levels of MC3R and MC5R in the patients’ tissue samples, positive responses were observed for MC3R in all patients and for MC5R in 84% of the patients. Interestingly, the expression of MC3R and MC5R was significantly higher in inflamed mucosa compared to normal mucosa. MC3R expression was 7.7 times higher in CD and 12 times higher in UC in inflamed mucosa, while MC5R expression was 5.5 times higher in CD and 8.1 times higher in UC in inflamed mucosa. Based on these results, the authors suggested that MC3R and MC5R are more highly expressed in regions of the large bowel with histological damage in IBD, which can be correlated with disease activity [216].

Based on several studies to date, the melanocortin system appears to be related to inflammation in IBD and is promising as both a diagnostic marker and a therapeutic target.

#### 3.1.11. Urotensin II and Urotensin II Receptor

Urotensin II (UII), a potent vasoactive peptide with vasoconstrictive action, has a strong affinity for its receptor (UTR) and is known to be distributed in most tissues throughout the body. UII exerts wide-ranging effects on various organs through UTR by inducing smooth muscle cell proliferation, collagen synthesis, and calcium mobilization [217,218].

Recently, it has been discovered that UII is involved in immune regulation [219], and studies have been conducted to investigate its association with IBD. In a pilot study by Gravina et al., UTR expression in colonic mucosa was found to be higher in UC patients compared to healthy controls, suggesting that UTR could be considered a disease marker for UC [220]. A subsequent study involving 26 patients with CD and 24 patients with UC showed that the concentration of UII was significantly higher in patients with IBD compared to the control group. However, no significant difference in UII concentration was observed between patients with CD and patients with UC. This study also demonstrated that serum UII levels positively correlated with high-sensitivity CRP, blood pressure, the UC Endoscopic Index of Severity, and the Simple Endoscopic Score for CD while showing a negative correlation with total proteins [217]. This implies that there is a direct association between UII levels and disease severity in IBD patients. Furthermore, a recent study including 100 UC patients revealed significantly higher UTR expression in the lesioned mucosa of UC patients and demonstrated a direct correlation between UTR expression and disease severity. Additionally, it was shown that UTR expression was associated with steroid treatment response: UTR expression was higher in the 72 patients requiring intravenous steroid therapy, with the 32 steroid non-responders exhibiting significantly higher UTR expression compared to the 40 steroid responders [221].

UII and UTR have shown promising results for the diagnosis and severity assessment of IBD, as well as for predicting therapeutic responses in IBD, suggesting their potential as clinically useful markers.

#### 3.1.12. Aquaporin System

Aquaporin (AQP) is a family of protein channels that form pores in the cell membrane, facilitating the movement of water, glycerol, and small solutes between cells [222,223]. Mammalian cells express 13 types of AQPs, and their distribution and permeability to substances vary depending on the type of AQP and the part of the body in which they are found [224].

Several studies have suggested a connection between AQPs and IBD. In a study conducted by Hardin et al., a significant decrease in AQP expression was observed in active UC, CD, and infectious colitis, which appeared to be related to disease severity [225]. A subsequent study including 22 patients with CD, 10 patients with UC, and 11 non-IBD controls found that AQP1, AQP3, AQP7, and AQP8 mRNAs were present in all parts of the intestine, with a notable decrease in AQP expression in both CD and UC. Furthermore, the levels of AQP mRNAs decreased differently in CD and UC [224]. The reduction in AQP expression can lead to restricted water reabsorption, potentially causing diarrhea. The decrease in AQP expression with the onset and worsening of IBD severity can be understood in this context [226,227]. Thus, AQPs have the potential to be used as markers of IBD.

### 3.2. Microbiome

In individuals with a genetic predisposition, an inappropriate immune response to gut microbiomes is thought to be part of the pathogenesis of IBD. Consequently, methods targeting the gut microbiome, such as probiotics, prebiotics, and fecal microbiota transplantation, have been researched as treatments for IBD, and there are studies attempting to utilize the microbiome for IBD diagnosis [228].

IBD patients have a different gut microbiota compared to healthy controls or IBS patients. Indeed, IBD patients show a lower abundance of *Faecalibacterium prausnitzii* compared to IBS patients and healthy controls [229]. In pediatric IBD, CD patients have fewer *Bifidobacterium* species compared to UC patients, and IBD patients requiring biological therapy have fewer butyrate-producing bacteria [230]. Zhou et al. developed a prediction model based on the gut microbiome, which demonstrated a prediction accuracy of 87.5% for CD and 79.1% for UC [231].

In addition to the diagnosis of IBD, the gut microbiome is associated with disease activity, treatment efficacy prediction, and prognosis [228]. Clearly, the gut microbiome is considered a highly useful marker in IBD. However, the analysis of the microbiome requires substantial costs and equipment, and previous studies have limitations such as a lack of reproducibility of the microbiota, thus limiting its routine use.

### 3.3. Endocytoscope and Confocal Laser Endomicroscopy

The endocytoscope can magnify lesions up to 450–1400 times, allowing observation at the cellular level to evaluate pit structure, microvasculature, and the degree of inflammatory cell infiltration [232]. In UC, endoscopic scores using the endocytoscope correlate well with disease activity and histology. Lacucci et al. developed the endocytoscopic score, which demonstrated a strong correlation with the Robarts Histopathology Index (RHI) (r = 0.89; 95% CI, 0.51–0.98) and the Nancy Histological Index (NHI) (r = 0.86; 95% CI, 0.42–0.98) [232]. The combination of the endocytoscope and narrow-band imaging (NBI) demonstrated excellent performance in diagnosing acute inflammation with sensitivity, specificity, PPV, NPV, and accuracy of 84.0%, 100%, 87.1%, 100%, and 92.3%, respectively, showing significant superiority in diagnostic specificity, NPV, and accuracy compared to conventional endoscopy [233]. Nakazato et al. also reported that the endocytoscopic score had a sensitivity of 0.77 and a specificity of 0.97 for histologic remission of UC [234]. A prospective study involving 32 patients with mild to moderate UC divided the patients into four groups based on endocytoscopic findings of pits and crypts. The study showed that structural deformities of pits and crypts were associated with recurrence [235]. Endocytoscopes are currently used predominantly in tertiary centers and are not yet utilized for daily use. However, its significant advantage of allowing histological evaluation of lesions without a biopsy indicates that it can play a major role in the diagnosis and assessment of IBD in the future.

Confocal laser endomicroscopy (CLE) provides information on functional abnormalities and dynamic changes in addition to the static information of conventional histology [236]. More than half of the patients who were in remission on white light endoscopy exhibited acute inflammation histologically in a prospective study on UC patients. Conversely, those who were normal or exhibited chronic inflammation on CLE did not show acute inflammation histologically [237]. This indicates that CLE can detect disease activity at the histological level that conventional endoscopy cannot. In a prospective study involving 181 IBD patients, barrier healing observed on CLE was reported to predict major adverse outcome-free survival better than endoscopic and histologic remission [238]. A meta-analysis reported a pooled sensitivity of 87% and a pooled specificity of 94% for CLE in distinguishing neoplastic lesions from non-neoplastic lesions in IBD patients [239]. In a randomized controlled trial that included 161 long-term UC patients in clinical remission, endomicroscopy with chromoscopy detected 4.75 times more neoplasms than conventional colonoscopy (*p* = 0.005) [240]. However, as with the endocytoscope, the routine use of CLE in IBD patients is still challenging due to cost and technical issues.

### 3.4. Artificial Intelligence

The diagnosis and management of IBD are complex, making it difficult for clinicians to make decisions easily. The diagnosis of IBD does not rely on a single tool but is mostly determined through various tests combined with the clinician’s judgment. In addition to the diagnosis, making decisions about changing medications and evaluating a patient’s disease activity can often be challenging.

The introduction of AI is expected to significantly reduce the complexity of IBD diagnosis and management. AI can assist in interpreting results from endoscopy, pathology tests, and radiologic tests and help in clinical decision-making by integrating the collected test results. Mossotto et al. applied a machine learning model to endoscopic and histological data to classify pediatric patients with CD, achieving classification accuracies of 71.0%, 76.9%, and 82.7% when using only endoscopic data, histological data, and both combined, respectively [241]. Tone et al. differentiated UC, CD, and ITB using a random forest and convolutional neural network (CNN) through endoscopy, achieving a diagnostic precision for UC/CD of 0.97/0.65 with random forest and 0.99/0.87 with CNN [242]. Additionally, a recent study showed that applying machine learning to analyze gut microbiome data could aid in the diagnosis of IBD [243].

Endoscopic scoring systems are used to evaluate disease severity; however, these scores are subject to interobserver variability. In a past study, 58 gastroenterologists were asked to evaluate the Mayo endoscopic subscore (MES) and Rutgeerts score (RS) from colonoscopy images and videos of UC and CD patients, with the overall interrater agreement being only 0.47 for MES and 0.33 for RS [244]. It also takes a long time for an individual to achieve a high level of interpretive skills. AI can help reduce inaccuracies and subjectivity. Ozawa et al. developed a CNN-based computer-assisted diagnosis (CAD) system and evaluated its performance using a large endoscopic image dataset of patients with UC. This CNN-based CAD system exhibited excellent performance in distinguishing MES 0 and 1 from MES 2–3, with an area under the receiver operating characteristic curve (AUROC) of 0.98 [245].

Research has also been conducted using AI to review VCE. Interpreting VCE requires maintaining high levels of concentration for an extended period of time, which raises concerns about missing lesions due to decreased attention. Various studies have used AI to address this issue by developing several CNN models to accurately recognize VCE findings such as strictures or ulcers. Klang et al. used a CNN to accurately and quickly detect ulcers in VCE images of patients with CD (AUC 0.99, accuracy 95.4–96.7%) [246]. Klang et al. also utilized deep neural networks to classify strictures and nonstrictures in CE images of CD patients, with an average accuracy of 93.5% [247]. Aoki et al. compared two review processes: endoscopists alone and endoscopists reviewing images prescreened by a CNN system. They found that the latter significantly reduced interpretation time (*p* < 0.001) without a significant reduction in the detection rate of mucosal breaks [248].

Recently, histological remission is increasingly being considered a long-term treatment target for IBD, although it is not yet a strict requirement. Several histological scoring systems have been developed; however, they are not widely used in clinical practice. Similar to endoscopy, there is interobserver variability [249]. Gui et al. developed the Paddington International virtual chromoendoscopy scope (PICaSSO) Histologic Remission Index (PHRI) and used a CNN-based deep learning strategy to differentiate active from quiescent UC, with a sensitivity, specificity, and accuracy of 78%, 91.7%, and 86%, respectively [250].

The concept of real-time monitoring of patients with IBD remains challenging owing to technical, ethical, and privacy issues, preventing its widespread application. Technical advancements in AI are expected to be significant in the future. AI is expected to be increasingly integrated into daily clinical practice, playing a major role in real-time patient monitoring, precision medicine, and shared decision-making with patients [251].

## 4. Suggestions for Future Research

Current diagnostic techniques for IBD have significantly advanced compared to past diagnostic methods. However, effective diagnosis of IBD, determination of treatment strategies, and prognosis prediction remain major concerns. Researchers have proposed various promising biochemical markers and tools. Continuous research is needed to further solidify the usefulness of these markers and procedures, as well as explore new ones. Research is also necessary to explore methods for implementing new procedures in clinical practice. Despite the promising utility of the microbiome, its complexity limits its clinical application. In addition to the microbiome, areas such as predictive model development, genetics, and histology contain complexities that are difficult to manage with human cognition alone. With the rapid advancement of AI, its active usage in research fields makes data integration and utilization easier. An increased understanding of the microbiome is expected to provide further insights into the pathogenesis of IBD. The use of AI in real-time patient monitoring and precision medicine is also expected to have a significant impact.

## 5. Conclusions

IBD is a condition whose pathogenesis is not yet fully understood, and it lacks a single diagnostic tool, making it difficult to diagnose. IBD specialists diagnose based on a combination of patient history, endoscopy, laboratory tests, histopathologic tests, and imaging tests, making clinical judgment critical. While UC and CD share similarities in treatment approaches, they have distinct clinical features that necessitate different treatments, underscoring the importance of accurate diagnosis. Clinical physicians perform tests based on the hospital’s situation and available medical resources and refer patients to higher-level hospitals if necessary. Generally, in outpatient clinics, physicians take detailed patient histories, perform physical examinations, and conduct non-invasive laboratory tests and, if possible, endoscopy. UC often involves the rectum and typically presents with clear symptoms such as diarrhea and hematochezia. However, CD, which can involve the small bowel, often has vague symptoms and lesions that may not be visible with standard upper endoscopy or ileocolonoscopy, requiring careful attention. It is essential to be familiar with the characteristic findings of IBD on endoscopy and imaging tests. In small clinics primarily operating outpatient services, actively using IUS can be beneficial. Recently, with the rise of AI, its application in diagnostics, treatment, and monitoring has been researched, and advancements in related technologies are anticipated in the future. Although routine use is not yet achieved, many promising new diagnostic procedures have been proposed. Thus, the practical application of these procedures and the development of additional new procedures necessitate ongoing research.

## Figures and Tables

**Table 1:** Comparison of colonoscopy findings between ulcerative colitis and Crohn’s disease.

Characteristics	Ulcerative Colitis	Crohn’s Disease
Pattern of ulcer [95]	Diffuse mucosal inflammation or ulceration	Transmural ulceration
Terminal ileal involvement [90,91,95]	Rare (back-wash ileitis)	Frequent
Rectal involvement [90,91,92,95]	Almost always	often spared
Continuous lesion [90,91,94,95]	Always	Infrequent
Skip lesion [90,94]	Rare	Frequent
Stricture [95]	Rare	Frequent
Anal or perianal disease [90,94,95]	No	Frequent
Fistula [95]	No	Frequent

**Table 2:** Comparison of differences in colonoscopy characteristics between intestinal tuberculosis and Crohn’s disease.

Characteristics	ITB	CD
Ulcer [95,96]	Circumferential or transverse (inflammed adjacent mucosa)	Longitudinal (normal adjacent mucosa)
Involved segments [95,96]	<4 segments	≥4 segments
Involvement of ileocecal valve [95,96]	Usually (patulous ileocecal valve)	Common
Anal or perianal disease [95]	Rare	Frequent
Stricture and fistula [97,98]	Rare	++
Cobblestone appearance [95,97]	+	++
Aphthous ulcer [95,96]	+	++
Pseudopolyp [95,96]	++	+

Abbreviations: ITB: Intestinal tuberculosis; CD: Crohn’s disease. Explanations: +: less likely to suggest; ++: more likely to suggest.

## Data Availability

Not applicable.
